# Eliminating malaria vectors

**DOI:** 10.1186/1756-3305-6-172

**Published:** 2013-06-07

**Authors:** Gerry F Killeen, Aklilu Seyoum, Chadwick Sikaala, Amri S Zomboko, John E Gimnig, Nicodem J Govella, Michael T White

**Affiliations:** 1 Environmental Health & Ecological Sciences, Ifakara Health Institute, Dar es Salaam, United Republic of Tanzania; 2 Vector Biology Department, Liverpool School of Tropical Medicine, Liverpool, UK; 3 National Malaria Control Centre, Lusaka, Zambia; 4 Monitoring & Evaluation Thematic Group, Ifakara Health Institute, Dar es Salaam, United Republic of Tanzania; 5 Division of Parasitic Diseases, Centers for Disease Control and Prevention, Chamblee, Georgia, USA; 6 Department of Infectious Disease Epidemiology, Medical Research Council Centre for Outbreak Analysis & Modelling, Imperial College London, London, UK

**Keywords:** Plasmodium, Control, Anopheles, Mosquito, Eradication, Elimination, Resistance, Behaviour

## Abstract

Malaria vectors which predominantly feed indoors upon humans have been locally eliminated from several settings with insecticide treated nets (ITNs), indoor residual spraying or larval source management. Recent dramatic declines of *An*. *gambiae* in east Africa with imperfect ITN coverage suggest mosquito populations can rapidly collapse when forced below realistically achievable, non-zero thresholds of density and supporting resource availability. Here we explain why insecticide-based mosquito elimination strategies are feasible, desirable and can be extended to a wider variety of species by expanding the vector control arsenal to cover a broader spectrum of the resources they need to survive. The greatest advantage of eliminating mosquitoes, rather than merely controlling them, is that this precludes local selection for behavioural or physiological resistance traits. The greatest challenges are therefore to achieve high biological coverage of targeted resources rapidly enough to prevent local emergence of resistance and to then continually exclude, monitor for and respond to re-invasion from external populations.

## Introduction

Proven implementation models exist for effectively achieving, validating and sustaining the elimination of malaria from areas with low transmission levels, predominantly relying upon drug therapy of residual human infections and careful surveillance with reliable diagnostic tests [1]. However, such conditions only occur at the margins of the current geographic range of malaria transmission and can only be attained in the most staunchly endemic parts of Africa and the Pacific by suppressing vectorial capacity by four orders of magnitude [2]. The primary obstacle to global malaria eradication remains the parasite’s historical strongholds in Africa and the southern Pacific, where unusually efficient vectors saturate human populations with intense transmission that dramatically attenuates, and may even negate, the impacts of drugs and vaccines [2-9]. In addition to eliminating blood and liver-stage parasites from local human populations and limiting reintroduction from external endemic areas, malaria elimination programmes in such historically endemic countries will also have to aggressively suppress the transmission potential of such potent vectors indefinitely unless global eradication of anthroponotic *Plasmodia* can be achieved [1,10]. It remains an open question as to whether it is possible to eliminate malaria transmission from settings with high climatic suitability for propagation of parasite sporogonic stages so long as even the sparsest populations of highly efficient anthropophagic vectors such as *Anopheles gambiae* persist [11,12]. While anophelism without malaria [13,14] has been achieved in several settings with either modestly efficient vectors or marginal climatic suitability for transmission, we are not aware of any example in which malaria transmission has been eliminated from any setting where the most anthropophagic and efficient vectors, such as *An*. *gambiae*, *An*. *funestus* or *An*. *punctulatus*, coincide with climatic conditions supportive of endemic, stable transmission. Genetic modification or population suppression strategies have been appropriately emphasized as options for eliminating mosquitoes and malaria [15] and some recent successes are particularly encouraging [16]. However, here we review examples of how insecticide-based approaches have successfully eliminated vector species in several tropical settings and explain how they might be extended to attack a much wider variety of target species if appropriate new vector control technologies were made available.

## Review

### The dependence of primary malaria vectors upon humans and livestock

Many of the world’s mosquito species, notably the most competent *Anopheles* vectors of human malaria, have highly selective host preferences and correspondingly adapted feeding behaviours [17,18]. Most of the world’s malaria burden occurs in sub-Saharan Africa because of three endemic species of highly specialized mosquitoes that almost exclusively rely upon humans (*An*. *funestus* and *An*. *gambiae*), or upon humans and their cattle (*An*. *arabiensis*), for blood [19]. These exceptional vector species can mediate intense malaria transmission levels, more than four orders of magnitude in excess of that required to sustain stable endemic populations of the *Plasmodium falciparum* parasite [2,20]. However, this dependence upon humans and their livestock also represents the Achilles’ heel of this disproportionately important trio of mosquito species [21], which can be exploited to render them locally extinct.

Insecticide-treated nets (ITNs) and indoor residual spraying (IRS) can achieve community-wide malaria transmission suppression by two orders of magnitude in African settings [22,23], vastly in excess of that attributable to direct coverage and personal protection [24,25]. The level of positive externality achieved by ITNs and IRS is exceptional among public health interventions and arises from (i) the heavy reliance of primary African vectors upon humans for blood, and (ii) the lengthy sporogonic incubation period of the malaria parasite inside the mosquito, during which time it may repeatedly risk fatal insecticide exposure while feeding or gestating every few days [24,25]. However, even high demographic coverage of humans (C_h_) with these measures rarely achieves elimination of malaria because vectors evade insecticide contact by feeding outdoors or upon cattle, thus creating gaps in biological protective coverage of the available blood resources (C_A,p_) that they rely upon [26]. Careful examination of where these gaps exist, how they might be closed, and what might be possible if they were closed, strongly suggests that all three of the species could well be eliminated and then excluded from large tracts of Africa.

### Primary vectors can be eliminated with imperfect interventions: evidence for Allee effects in mosquito populations?

In the most extreme examples, IRS and ITNs have selectively eliminated the most efficient local primary vectors because the strict dependence upon humans that make them such potent agents of transmission also renders them vulnerable to these two means for delivering insecticides to houses and sleeping spaces [21,26]. For example, the notoriously efficient, anthropophagic, endophagic and endophilic species *An*. *funestus* disappeared entirely from the Pare-Taveta study area in Tanzania after three years of IRS with dieldrin [27,28] and took five years to detectably re-establish itself in the area following the cessation of spraying [29]. It is fundamentally difficult to prove the absolute absence of malaria parasites [10] and the same is true of their vectors. However, it is notable that when *An*. *funestus* did re-appear, the spectacular speed at which it rebounded to exceed its pre-intervention population size [29] does suggest that it had indeed been truly absent for several years after spraying had ceased. Similarly, *An*. *funestus* essentially disappeared from Malindi on the coast of Kenya following the introduction of IRS with DDT [30]. In Malindi and in south Pare, but curiously not in Taveta, *An*. *funestus* appears to have been replaced by increasing numbers of zoophagic, exophagic and exophilic *An*. *rivulorum* and *An parensis* from the same species group, presumably as a result of a shift in the balance of competition for limiting aquatic habitat resources [27,28,30,31]. *An*. *funestus* was also eliminated from almost the entire country of South Africa using IRS with DDT in the 1950s [32]. With the exception of one observation, both the vector and the malaria transmission it mediated remained absent for four decades, only to return when DDT was replaced with pyrethroids, against which resistance rapidly emerged [33]. Following careful susceptibility surveys of this invasive population, DDT was re-introduced and IRS was extended to a regional programme that achieved or closely approached local extinction of this species [34]. Similarly, *An gambiae* has proven vulnerable to IRS in Nigeria during the Global Malaria Eradication Programme (GMEP) [35], and to ITNs in contemporary Kenya and Tanzania where it has become very scarce in several settings [36-38]. In the Pacific, *An*. *koliensis* has been eliminated from the Solomon Islands by a series of IRS and ITN campaigns over the last 4 decades [39] and was last seen on Malaita in 1987 [40]. *An*. *punctulatus* is now only patchily distributed across a fraction of its former range within the archipelago [39]. In Latin America, historical accounts from Guyana of *An*. *darlingi* elimination with IRS [41] are now complemented by contemporary evidence of local extinction of this same species, as well as *An*. *nuneztovari*, following ITN scale up in neighbouring Suriname [42].

**Figure 1:**
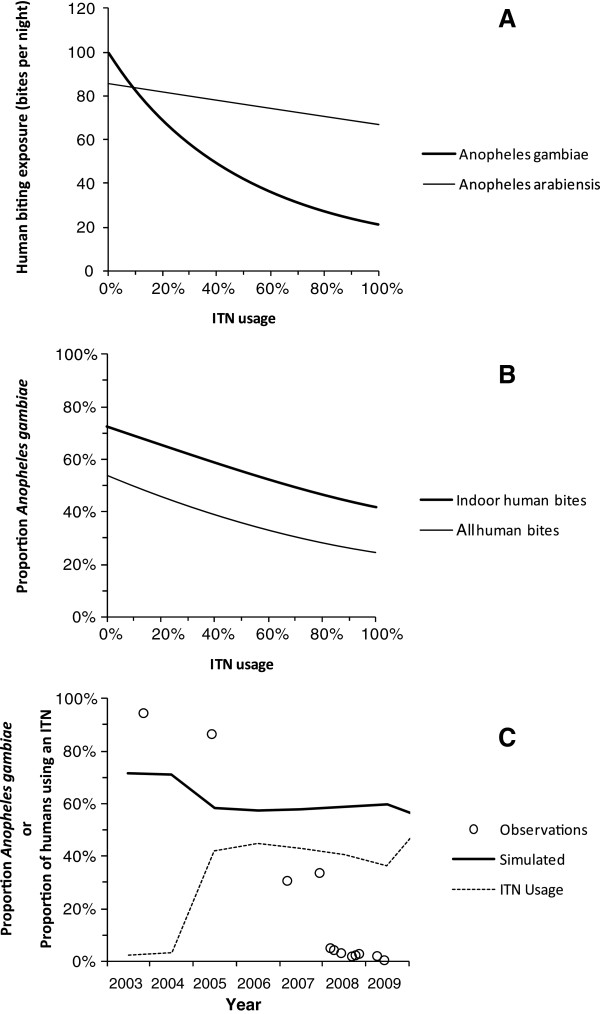
**Contrasting field observations of the collapse of *An***. ***gambiae *with the predictions of population dynamics models assuming no density**-**dependence of vector reproduction so that emergence rates are directly proportional to mean longevity. A**: Simulated declining biting exposure to *Anopheles gambiae s*.*s*. and *An*. *arabiensis* as insecticide treated net usage (ITNs) increases. **B**: Corresponding impact of increasing ITN use upon predicted proportion of bites upon humans by *An*. *gambiae*. **C**: Direct comparison of observed near-disappearance of *An*. *gambiae* from Kilombero Valley in southern Tanzania [37] with simulations based upon observed ITN usage rates estimated as described previously, with only 4.7% of nets treated within the previous 6 months up to 2004 [48], following which long-lasting net retreatment kits were introduced so all nets reported as treated are considered ITNs [49]. All simulations were executed [43] assuming equal baseline emergence rates (E_0_ = 2 × 10^7^ mosquitoes per year) for both vector species and a ratio of cattle to humans consistent with livestock census results in Kilombero Valley (N_h_ = 1000, N_c_ =140). All ITN-induced mortality was assumed to occur before feeding so the excess proportion of mosquitoes killed after attempting to attack a protected human was assumed to be negligible (θ_u, post_ = 0). Simulated *An*. *gambiae s*.*s*. and *An*. *arabiensis* populations differed only in their parameter values for the proportion of human exposure to bites that occurs indoors (π_i_ = 0.9 versus 0.4, respectively [37,50]), the attack availability rates of cattle (a_c_ = 2.5 × 10^-5^ versus 1.9 × 10^-3^ attacks per host per host-seeking mosquito per night [51]) and the excess proportions of mosquitoes which are diverted (θ_Δ_ = 0.2 versus 0.6, respectively) or killed before feeding (θ_μ,pre_ = 0.8 versus 0.6, respectively) while attempting to attack a human while using an ITN [52-54].

A common feature to all these historical examples of eliminating human-dependent mosquitoes with domestic applications of insecticides is that existing models of malaria transmission and mosquito population dynamics cannot explain them (Figure 1). Even recent models assuming the kind of linear dependence of emergence rates upon mean longevity [43] that would only be expected far below the carrying capacity of available larval habitat [44,45], predict the dominance of *Anopheles arabiensis* as a vector of residual transmission at high ITN coverage (Figure 1) but fail to capture the dramatic collapse of *An*. *gambiae* populations that is often observed in practice [36-38,46,47]. The predicted reductions of both *An*. *arabiensis* and *An*. *gambiae* biting densities that are depicted in Figure 1A make intuitive sense based on the input parameters for mosquito human feeding preference, indoor-biting propensity, diversion away from attack, and attack-associated mortality that were assumed based on detailed field study of these species, but fall far short of what is usually expected from ITNs or IRS in Africa based on the extensive contemporary empirical evidence base [22,23].

Local elimination of malaria vectors with imperfect interventions has also been achieved with strategies other than the adulticide-based interventions prioritized today [55]. There are several historical records of eliminating African primary vector species from substantial tracts of Brazil, Egypt and Zambia, primarily through larval source management of aquatic habitats [56-60]. Beyond malaria vectors, the Australian vector of Ross River Virus, *Aedes camptorhynchus*, has been eliminated from New Zealand where it was an invasive exotic, primarily through larvicide treatment of salt marshes [61]. Perhaps one of the greatest tragedies in public health today is that the elimination of *Aedes aegypti* from most of Latin America, primarily through larval source management, by the early 1960′s [62] was not extended or even sustained, so this species remains the most globally important vector of Dengue, Yellow Fever and Chikungunya viruses today.

These remarkably numerous examples of vector population collapse are surprising because their reproduction rates are now known to be primarily limited by aquatic habitat availability. This suggests that reductions in the number of adult mosquitoes emerging from breeding sites due to control measures that reduce numbers of adults should be attenuated by density-dependent regulation at the aquatic stage, leading to increased *per capita* survival of the larval stages and even increased maternal fitness of the resulting adults [44,45].

**Figure 2:**
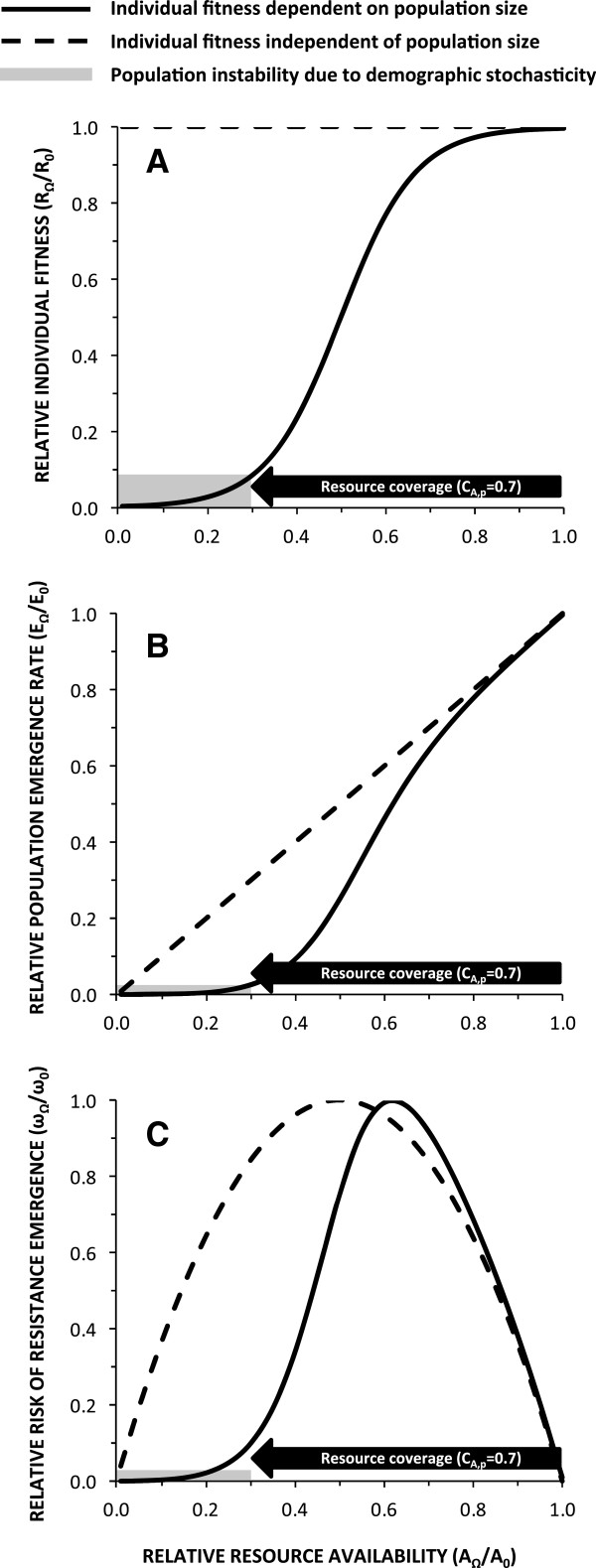
**A schematic illustration of how the dependence of individual fitness (A) upon total population size or density [**[63]**] (B) could hypothetically cause mosquito populations to collapse without the opportunity for resistance to emerge (C) following rapid deployment of insecticidal vector control measures at high but imperfect levels of biological coverage.** All outcomes plotted along the Y axis represent relative values for a given intervention scenario (Ω), with a given level of protective coverage of all available blood, sugar or aquatic resources (C_A,p_), compared to a baseline scenario (Ω = 0). **A**: Individual fitness is expressed as the reproductive number (R; the average number of female adult offspring per healthy female adult mosquito). **B**: Population size is expressed as the adult emergence rate (E). **C**: The rate at which the frequency physiological insecticide resistance traits increase to fixation or equilibrium.

We therefore hypothesize that mosquito populations are subject to strong demographic Allee effects, meaning that fitness of individuals is compromised at low population sizes or densities [63,64]. Allee effects generally occur when populations become so sparse that males and females struggle to find each other or when co-operative behaviours are disrupted [63,64]. Such a small insect commuting distances of up to 10km between aquatic habitats and human blood sources [65,66] probably experiences considerable stress when mating opportunities are restricted. Also, aggregation of males into swarms, within which they compete for females, is a co-operative behaviour that directly precedes the mating event itself. It is, therefore, easy to envisage how such Allee effects would amplify the effects of control measures as they are increasingly effective. Extinction through demographic stochasticity could, therefore, be achieved with high but imperfect intervention coverage of the blood, sugar or aquatic habitat resources mosquitoes need. Figures 2A and B illustrate how this phenomenon might hypothetically enable 100% elimination of a local mosquito population with only 70% biological coverage [26] of an effective intervention. While Allee effects are difficult to demonstrate at a demographic-level, individual-level effects upon fitness components, and mechanisms to avoid them, are widespread among animal, plant and even microbe populations [63,64]. Most mosquito populations go through dramatic seasonal fluctuations in resource availability and population size so Allee effects are most likely to be manifested and enhanced by vector control during the depths of the dry season. The seasonal contraction of aquatic habitat to very limited, focal refugia represents a recurring opportunity to eliminate them entirely using improved or additional vector control strategies [15,67] that need not necessarily be effective outside of these troughs of population size and habitat availability [56,68].

The possibility that vector populations exhibit Allee effects also has important, even more encouraging, implications for resistance management: The range of biological coverage levels at which resistance traits are rapidly selected for will be much narrower and the range of high coverage levels at which local elimination occurs will preclude local selection (Figure 2C). Aggressive, rapidly scaled up intervention packages may well be able to win the evolutionary arms race by simply extinguishing mosquito populations faster than they can adapt to intervention pressure.

### Intervention coverage of resources that are essential to mosquito survival: Closing the gaps

So if malaria vector populations can be eliminated with incomplete coverage of vector control interventions, why are such encouraging examples the exceptions rather than the rule? For larval source management strategies, the cryptic nature and unpredictable distribution of many aquatic habitats is often problematic but the primary obstacle to success is usually the massive logistical challenge of achieving high habitat coverage in practice [69,70].

**Figure 3:**
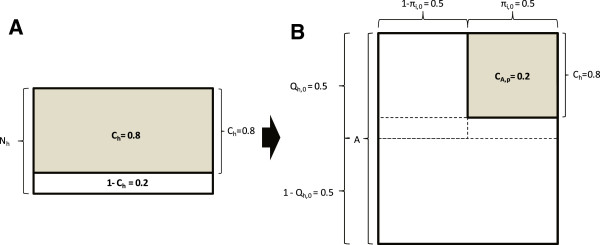
**Conceptual schematic of the difference between current demographic indicators of coverage of all humans (N**_**h**_**) and true biological coverage of all available mosquito blood resources (A) **[26]**.** In all panels, the proportion considered covered by the stated indicator is represented by the shaded fraction. **A**: Conventional view of current ITN/IRS target of 80% crude demographic coverage of all humans while indoor (C_h_ = 0.8). **B**: Biological protective coverage (C_A,p_ = 0.2) of all available human and animal blood (A) achieved in the same demographic coverage scenario (C_h_ = 0.8), where half of baseline human exposure to vectors occurred outdoors (π_i,0_ = 0.5) and animals previously accounted for half of all blood meals (Q_h,0_ = 0.5).

**Figure 4:**
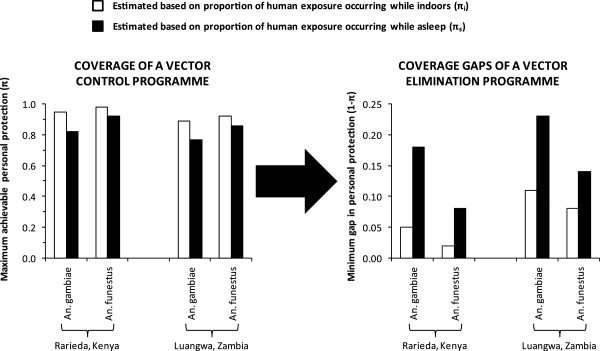
**Comparative evaluation of two alternative measures of the upper limit for the level of protection an insecticide treated net (ITN) can provide from the contrasting perspectives of coverage achieved by a vector control programme versus coverage gaps in a vector elimination programme.** The proportion of exposure to mosquito bites which occurs while humans are indoors (π_i_) overestimates protective coverage and underestimates gaps in protective coverage when compared with the proportion of exposure occurring while asleep (π_s_), which is considered a more accurate indicator of protective coverage by an ITN because people rarely use one unless they are both indoors and asleep [77].

For vector control measures targeted at humans, high demographic coverage of humans has clearly underpinned all the most impressive examples of vector elimination [27,28,30,35-37,39,41,42]. However, all these successes also relate to the most extremely human-dependent of all mosquito species, with feeding and resting habits which render them particularly vulnerable to IRS and ITNs. Such high behavioural vulnerability means that the biological coverage of all available blood and resting site resources (C_A_) closely approximates to simple demographic coverage [26], surveyed in the field as the proportion of human individuals or households directly protected (C_h_). These extreme examples of selective control are all nicely framed in the context of far less dramatic impacts upon less vulnerable species or molecular forms in the same settings, such as the M form of *An*. *gambiae*[71], *An*. *arabiensis*[35-38,47,52,53,72], *An*. *rivulorum* and *An*. *parensis*[27,28,30,31] and even previously undescribed species [73] in African settings, as well as *An*. *farauti* in the Solomon Islands [39]. As illustrated in Figure 3, all these species persist in the face of high demographic coverage with ITNs or IRS, in approximate proportion to their ability to attenuate biological coverage [26] by obtaining blood from humans outside of houses and from common alternative hosts such as cattle [4,21,74-76]. Simply aiming to eliminate, rather than merely control vectors prompts insightful consideration of differences between coverage gaps rather than coverage itself [77]. What might seem a minor difference between two different estimates of coverage may in fact disguise very large differences between corresponding estimates for the coverage gaps that allow vector populations to survive and transmit malaria (Figure 4).

Vectors with low behavioural vulnerability to IRS and ITNs have historically mediated the bulk of transmission across most of the modestly endemic regions of tropical Asia and Latin America [19,78-80]. Furthermore, they can also dominate residual transmission in previously holoendemic parts of the tropics where ITNs and/or IRS are maintained at high coverage [38,39,71]. A recent report suggests that an important, predominantly exophagic, novel vector species in the highlands of Kenya may have been systematically overlooked due to methodological limitations of standard morphological taxonomy [73] and further examples may be revealed in other African settings upon closer inspection with appropriate molecular techniques.

However, understanding where these limitations of existing intervention measures lie (Figures 3 and 4), and being aware of the apparent instability of vector populations when high biological coverage is achieved (Figures 1 and 2), provides grounds for optimism that a far wider range of malaria vectors in settings all across tropical Africa, Asia and Latin America can be eliminated if products targeted towards very different mosquito life stages and behaviours can be developed. If such behaviourally-mediated gaps in biological coverage of the ecological resources utilized by mosquitoes (Figure 3) could be closed by using interventions that target mosquitoes at source, or while resting and feeding upon humans or livestock outside of houses [15,26,67,75,76], it may well be possible to eliminate even these elusive primary vectors. Indeed, setting vector elimination as a deliberate target even enables complementary approaches to be rationally combined in ways that would not otherwise make sense. For example, if biological coverage and impact of adult control can be dramatically improved, it may be possible to apply supplementary larval source management in a time-limited manner during the dry season when this strategy is far more practical and affordable, to not only eliminate parasite transmission [2] but also to extinguish residual foci of vector proliferation when they are most vulnerable [56-60].

### Programmatic and biological sustainability of vector elimination strategies

The intimidating technological and programmatic obstacles to adopting, developing and implementing vector elimination strategies are obvious and merit careful consideration. Perhaps the greatest challenge facing vector elimination initiatives will be to maintain buffer zones along their periphery with even more aggressive, diverse and sustained intervention packages which, not only prevent re-invasion through immigration, but also preclude emergence of insecticide-resistant vector populations at this transitional interface between stable source and unstable sink populations [81]. The qualitative and quantitative diversity of this challenge is strongly influenced by local physical and human geography. While geographic fragmentation of the Pacific region into thousands of islands might seem ideally suited to the technical requirements of parasite or vector elimination programmes, this situation exacerbates the operational challenges facing successful implementation [82]. Furthermore, such fragmentation and isolation also generates spectacular biodiversity [83], including a bewildering array of *Anopheles* species [84], many of which exhibit considerable structure with local populations [85]. Perhaps the greatest challenge is Africa where operational constraints are extreme [82] and the sheer size of the continent has always buffered the continent against mass extinctions [86].

While these challenges should not be dismissed lightly, neither should the potential benefits of addressing them. The immediate public health benefits of eliminating the most potent malaria vectors are obvious, and well documented in several instances [34,87-90], but there are also several advantages in terms of the financial, logistic and biological sustainability of such an ambitious strategy. The fundamental advantage of any elimination strategy for malaria parasites [91] or vectors [56,57] is that it is time-limited in any given location, except for barrier areas on the periphery of a protected zone, country or region. Far more aggressive, ambitious intervention packages can therefore be applied over finite times and places than would be possible to sustain universally and indefinitely.

Biologically, the major advantage of a vector elimination strategy is simply that non-existent populations cannot develop physiological or behavioural resistance. Recent theoretical analysis suggests that physiological resistance traits will most rapidly emerge in malaria vector populations where coverage with ITNs is incomplete or patchy, corresponding to the peaks in the middle of Figure 2C [92]. The greatest danger to our most valuable insecticides may therefore lie in control programmes that achieve only mediocre coverage for extended periods. Interestingly, this analysis also considered the outdoor environment as a refuge [92] for exophagic mosquitoes (Figure 3), so the behavioural characteristics of all targeted vectors in a given area must be actively measured and carefully considered in any resistance management scheme. Crucially, it is often the innate flexibility of behaviours that allows many species of mosquitoes to immediately cope with the changes of resource availability in their environment that intervention scale up represents [18,93-95]. Beyond mosquitoes, such phenotypic plasticity is now known to allow a wide variety of organisms to not only immediately survive otherwise stressful conditions but also to evolve further heritable adaptive traits in the longer term [96-98].

Much of the agricultural pest literature suggests that coverage gaps or refugia will delay the emergence of resistance [99] and this easing of selection pressure is represented by the downward slopes on the right hand side of the peaks in Figure 2C. However, assuming that the rate at which selectable resistance traits arise is dependent upon absolute population size, the risk of such traits emerging cannot increase monotonically with increasing intervention pressure and decreasing population size. Resistance emergence risk must reach a peak where the interaction between selection pressure and population density is maximized, and then decreases to zero as intervention coverage increases and population size shrinks. Reaching the downward slope on the left hand side of these peaks therefore represents an equally viable way to reduce the risk of resistance emergence, especially if Allee effects enable local population extinction within manageable ranges of high but imperfect coverage (Figure 2C).

## Conclusions

Human-dependent mosquito populations can rapidly collapse when they are forced below realistically achievable, non-zero thresholds of density and supporting resource availability. Local population elimination may be achievable for a much wider range of malaria vectors if insecticidal vector control tools can be developed which cover a wider range of the blood, resting site, sugar and aquatic habitat resources they need to survive [15,67]. Perhaps the greatest challenge facing vector elimination initiatives will be to maintain buffer zones with even more aggressive intervention packages which prevent re-invasion and emergence of insecticide-resistant traits at this transitional interface. Nevertheless, the potential long term benefits of embracing such an ambitious strategy merit strategic consideration: In addition to achieving complete local control of such dangerous insects across large geographic areas, aggressive vector elimination campaigns also offer a temptingly direct resistance management strategy as an alternative to repeated rotation cycles of constantly changing insecticide combinations or mosaics, commonly referred to in agriculture as the “insecticide treadmill”.

## Abbreviations

GMEP: Global Malaria Eradication Programme; IRS: Indoor residual spraying; ITN: Insecticide-treated net.

## Competing interests

The authors declare that they have no competing interests.

## Authors’ contributions

GFK reviewed the literature and formulated the hypotheses with MTW. GFK wrote the paper with assistance from AS, CS, JEG, NG and MTW. All authors read and approved the final manuscript.
